# Galectin-3 interacts with components of the nuclear ribonucleoprotein complex

**DOI:** 10.1186/s12885-016-2546-0

**Published:** 2016-07-19

**Authors:** Katharina Fritsch, Marco Mernberger, Andrea Nist, Thorsten Stiewe, Alexander Brehm, Ralf Jacob

**Affiliations:** Department of Cell Biology and Cell Pathology, Philipps-Universität Marburg, Robert-Koch-Str. 6, D-35037 Marburg, Germany; Institute of Molecular Oncology, Philipps-Universität Marburg, Marburg, Germany; Genomics Core Facility, Philipps-Universität Marburg, Marburg, Germany; Institute for Molecular Biology and Tumor Research, Philipps-Universität Marburg, Marburg, Germany

**Keywords:** Galectin-1, Galectin-3, hnRNPA2B1, RNA-processing, Spliceosome

## Abstract

**Background:**

The multifunctional β-galactoside-binding protein galectin-3 is found in many distinct subcellular compartments including the cell nucleus. Expression and distribution of galectin-3 between the cell nucleus and the cytosol changes during cell differentiation and cancer development. Nuclear functions of galectin-3 and how they contribute to tumorigenesis are not understood.

**Methods:**

In order to identify nuclear galectin-3 interaction partners, we used affinity chromatography and co-immunoprecipitation. Spatial proximity in the nucleus was assessed by immunofluorescence and proximity ligation assay. We also investigated the function of galectin-3 on mRNA-export by fluorescence *in situ* hybridization and on mRNA-processing by RNA-sequencing.

**Results:**

The heterogeneous ribonucleoprotein particle component hnRNPA2B1 was identified as a novel galectin-3 binding protein that associates with the lectin in a lactose-dependent manner in the cell nucleus. Specific individual depletion of galectin-3 does not affect the mRNA distribution between cytoplasm and nucleus. A significant alteration of this distribution was observed after combined depletion of galectin-1 and −3. However, silencing of galectin-3 was sufficient to alter the splicing patterns of several genes.

**Conclusions:**

Galectin-3 and hnRNPA2B1 interact as members of the early splicing machinery. Galectin-3 and −1 have redundant functions in mRNA transport and at least in part in mRNA splicing. RNA-sequencing data points to a specific function of the hnRNPA2B1/galectin-3 interaction in the processing of transcripts coding for the nuclear oncoprotein SET.

**Electronic supplementary material:**

The online version of this article (doi:10.1186/s12885-016-2546-0) contains supplementary material, which is available to authorized users.

## Background

The galectins are a family of small soluble sugar binding proteins characterized by a carbohydrate recognition domain (CRD). This CRD shows a conserved sequence motif and has a high affinity for β-galactosides [1]. The family comprises 15 mammalian galectins with one or two CRDs. Part of the lectin family is distributed in many different cell types (galectin-1, galectin-3, galectin-8, galectin-9), while galectin-2, galectin-4 and galectin-7 show a more restricted distribution. According to their domain composition, galectins have been classified into three subgroups, the prototype, the tandem repeat and the chimeric type. Prototype and chimeric type galectins contain one single CRD, whereas tandem repeat galectins are composed of two CRDs. Galectin-3 is the sole chimeric type galectin. It is composed of a proline- and glycin-rich amino-terminal domain fused to a carboxy-terminal CRD. Galectin-3 can be detected intracellularly in transport vesicles, the cytoplasm and the nucleus as well as in the extracellular milieu [2, 3]. The subcellular distribution of galectin-3 depends on the cell type and the proliferation stage [4–6]. This protein is involved in a large number of physiological and pathological processes such as cell proliferation, differentiation, survival, apoptosis, intracellular trafficking and tumor progression [7, 8]. Galectin-3 expression changes with tumor category. In cancers of the thyroid, liver, stomach, and central nervous system the protein is upregulated, whereas in cancers of the breast, ovary, uterus and prostate galectin-3 is downregulated (reviewed in [9]). Moreover, the subcellular distribution of galectin-3 varies among different tumor types. In tongue carcinoma cells galectin-3 is translocated from the nucleus to the cytoplasm during neoplastic progression [10]. In human colon and prostate carcinoma cells galectin-3 is generally down-regulated and consistently excluded from the nucleus [11]. On the other hand, in esophageal squamous cell carcinoma patients elevated expression of galectin-3 in the nucleus is a significant pathological parameter related to histological differentiation and vascular invasion [12].

Nuclear galectin-3 has a wide range of functions, one of them is the regulation of gene transcription. Galectin-3 promotes trans-activation functions of transcription factors CREB and Sp1, and induces cyclin D_1_ promoter activity in human breast epithelial cells [13]. Galectin-3 also modulates gene transcription by the interaction with the nuclear thyroid-specific transcription factor TTF-1 [14]. As transcriptional co-regulator, galectin-3 also binds Suppressor of fused, a negative regulator of the hedgehog signal-transduction pathway shuttling between the cytoplasm and the nucleus [15]. Another role of nuclear galectin-3 is its function as a pre-mRNA splicing factor. Early experiments already revealed that galectin-3 interacts with components of the nuclear ribonucleoprotein complex (hnRNP) [16]. Thereafter, a requirement for galectin-3 in pre-mRNA splicing was reported [17].

In our previous studies we observed an increase in nuclear translocation of galectin-3 in clear cell renal cell carcinoma cells [18]. To gain a better understanding of the nuclear functions of galectin-3 we now searched for putative galectin-3 binding partners in nuclear extracts and identified the heterogeneous ribonucleoprotein particle component hnRNPA2B1. Galectin-3 as well as hnRNPA2B1 co-localize in splicing factor enriched subnuclear speckles. Specific depletion of the two galectins, galectin-1 and −3, affects mRNA-export from the nucleus as assessed by fluorescence *in situ* hybridization. Single knockdown of galectin-3 alters the splicing patterns of several genes, including the SET-oncogene, which is also affected in hnRNPA2B1-depleted cells.

## Methods

### Antibodies, plasmid, siRNAs and oligos

Monoclonal (mAb) anti-galectin-3 (M3/38), mAb anti-galectin-3 (A3A12), mAb anti-galectin-1 (C-8) and polyclonal (pAb) anti-galectin-3 (H-160) antibodies were purchased from Santa Cruz Biotech, Dallas, U.S. MAb anti-Sc35, mAb anti-hnRNPA2B1 (DP3B3) and pAb anti-hnRNPA2B1 antibodies were obtained from Abcam, Cambridge, U.K. MAb anti-U2AF65 (MC3) and mAb anti-α-tubulin (DM1A) antibodies were purchased from Sigma-Aldrich, St. Louis, U.S. Secondary Alexa-coupled antibodies used for immunofluorescence were obtained from Invitrogen, Darmstadt, Germany.

Specific siRNAs 5’-CACGGTGAAGCCCAATGCAAA-3’ (NM_001177388) for galectin-3-depletion were purchased from Qiagen and siRNA sc-35441 for galectin-1-depletion was obtained from Santa Cruz Biotech, Dallas, U.S. For control experiments firefly luciferase-siRNA was used. Biotin-oligo(dT) for the FISH-assay was obtained from Eurofins MWG Operon, Ebersberg, Germany, and the secondary streptavidin-Alexa Fluor 546 antibody was purchased from Invitrogen, Darmstadt, Germany.

### Cell culture and transfection

Human cervix carcinoma cells (HeLa) were cultured in DMEM high glucose/10 % FCS, 2 mM glutamine, 100 U/mL penicillin, 100 mg/mL streptomycin. Human kidney clear cell carcinoma cells (RCC-FG1) were cultured in Mc Coy’s 5a/10 % FCS, 2 mM glutamin at 37 °C and high humidity. HeLa cells were transfected by electroporation with the Biorad Gene Pulser II. Up to 15 μg of siRNA were used for silencing of galectin-3 and/or galectin-1. For successful depletion the cells were transfected twice and harvested 48 h thereafter.

### Immunofluorescence, FISH and fluorescence microscopy

Fluorescence microscopy was performed with fixed HeLa cells essentially as described before [19]. Fluorescence in situ hybridization (FISH) was performed with fixed HeLa cells according to Chakraborty and Fontoura [20]. Cells were fixed with 4 % paraformaldehyde and permeabilized with 0.5 % Triton X-100 for 5 min at 4 °C. Pre-hybridization-mix (2 x SCC (3 M NaCl, 300 mM trisodium citrate, pH 7), 1 mg/mL tRNA, 10 % dextran-sulfate, 25 % formamide) was added to the cells and incubated for 15 min at 42 °C. The samples were then shifted to hybridization-mix (2 x SCC, 1 mg/mL tRNA, 10 % dextran-sulfate, 25 % formamide, 50 μg/mL Biotin-oligo(dT)) and incubated overnight at 42 °C followed by Streptavidin Alexa Fluor 546 incubation in PBS/ 0.2 % Triton X-100 for 30 min at room temperature. Confocal images were recorded on a Leica TCS SP2 microscope with a 40x objective (HCX PL APO CS 40x/1.25–0.75 oil), analyzed with LAS AF (Leica) and quantified with ImageJ.

### Proximity ligation assay

The *in situ* Proximity Ligation Assay (PLA) was performed by using the Duolink *in situ* kit purchased from Olink Bioscience. Cells were fixed with 4 % paraformaldehyde and permeabilized with 0.1 % Triton X-100 for 4 min at room temperature. The cells were blocked by adding blocking solution (Duolink) for 1 h at 37 °C. Monoclonal anti-galectin-3 A3A12 and polyclonal anti-hnRNPA2B1 were incubated overnight at 4 °C. Duolink *in situ* PLA probes anti-mouse PLUS and anti-rabbit MINUS were added and incubated for 1 h at 37 °C. Ligation-reaction and ligase were added followed by incubation for 30 min at 37 °C. Amplification was carried out for 100 min at 37 °C followed by fluorescence microscopy of the samples.

### Co-immunoprecipitation and BN-PAGE

Nuclear extracts (NE) from RCC FG1 cells and HeLa cells were prepared with NE-PER nuclear and cytoplasmic extraction reagents-kit obtained from Thermo Scientific, Dreieich, Germany. The buffer was changed to RIPA buffer (50 mM Tris–HCl pH 7.4, 150 mM NaCl, 1 mM EDTA, 1 % Triton X-100, 1 % sodium deoxycholate, 0.1 % SDS) by using Amicon Ultra-0.5 centrifugal filter units with ultracell-10 membrane (Merck Millipore, Schwalbach, Germany). For co-immunoprecipitation the Dynabeads M-280 sheep anti-rabbit IgG and the immunoprecipitation kit purchased from life technologies was used. NE were incubated with antibody-coupled Dynabeads for 20 min at room temperature. Samples were analyzed by SDS-Page and Western Blot. Blue native polyacrylamide gel electrophoresis (BN-PAGE) was performed essentially as described by Fiala and Blumenthal [21]. NE from HeLa cells were incubated in native sample buffer (50 mM BisTris, 125 mM 6-AcA, 0.1 % Triton X-100, pH 7.0). After 10 min 0.5 % coomassie brilliant blue G250 was added and incubated for 5 min at 4 °C. The samples were separated on 20 to 8 % native gradient gels. Second dimension was separated by 10 % SDS-PAGE and then transferred on a PVDF membrane for immunoblot.

### Protein purification, affinity chromatography and mass spectrometry

Recombinant human galectin-3 was isolated essentially as described before [22]. For the generation of affinity chromatography columns recombinant human galectin-3 was coupled on a HiTrap NHS-activated HP column (GE Healthcare) via primary amines. The column was washed with HCl (1 mM) and galectin-3 was circulated on the column for 60 min at 4 °C. Non-specifically bound ligands were removed with buffers A (0.5 M ethanolamine, 0.5 M NaCl, pH 8.3) and B (0.1 M sodium acetate, 0.5 M NaCl, pH 4). The column was equilibrated at room temperature followed by washing with buffers A, B and excess PBS. Putative interaction partners were eluted with elution buffer (150 mM lactose in PBS) and protein-rich fractions (absorption at 280 nm) were processed for mass spectrometry in MALDI-TOF or in MALDI-TOF-TOF mode, using a Voyager DE STR instrument (PerSeptive Biosystems, Framingham, USA) or an Ultraflex Instrument (Brucker, Germany).

### RNA-seq sample preparation and RNA-seq analysis

HeLa cells were transfected with siRNA to silence galectin-3 and with the non-silencing control siRNA against firefly luciferase. Total RNA was isolated with the RNeasy Mini Kit (Qiagen) and processed by the TruSeq® Stranded mRNA LT Kit to prepare RNA-seq libraries. The libraries were sequenced on an Illumina HiSeq 1500 sequencer via paired-end sequencing to obtain 2×50 bp paired reads. The obtained reads were mapped against the *Homo sapiens* genome reference (Ensemble Revision 74, hg19) using the STAR algorithm [23]. FPKM values were calculated for each sample, differential gene expression was analyzed using DEseq2 [24]. The genes with a FPKM value above 0.3 in at least one sample and a DEseq *p*-value of 0.05 or better were considered as differentially expressed if the absolute of the log2 fold change was one or larger. Differential exon usage was analyzed using DEXseq [25]. All algorithms used standard parametrization.

### Statistical analysis

Data are expressed as means SD and statistical significance was determined using an unpaired *t*-test with GraphPad Prism 5 (GraphPad Software, La Jolla, U.S.).

## Results

### Nuclear interaction of galectin-3 with hnRNPA2B1

**Fig. 1:**
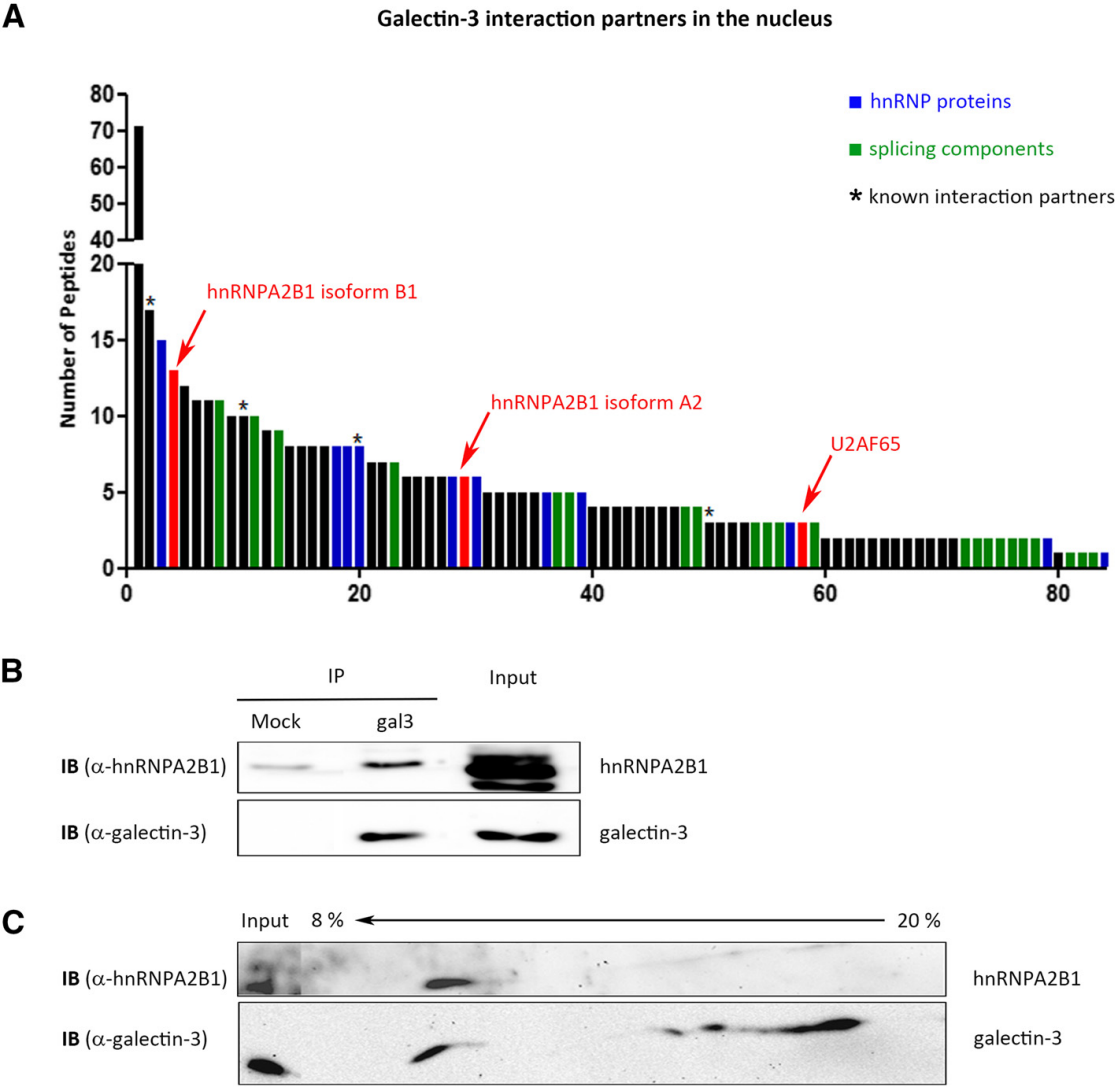
Interaction of galectin-3 with hnRNPA2B1 in nuclear extracts. hnRNPA2B1 was identified as an interaction partner of galectin-3 by affinity chromatography (**a**) and co-immunoprecipitation (**b**). **a** Interaction partners of galectin-3 were eluted with lactose from a galectin-3-coupled sepharose column and analysed by mass spectrometry. Among others, two isoforms of hnRNPA2B1 and the splicing auxiliary factor U2AF65 were identified as lactose-dependent interaction partners of galectin-3. Numbering on the x axis correlates with Additional file 1: Table S1. **b** Co-immunoprecipitation of hnRNPA2B1 with galectin-3. In “Mock” agarose beads control anti-antibodies were used as negative control. **c** Immuno blot analysis of the second dimension of a Blue Native PAGE of NE. The percentages of polyacrylamide in the first dimension under native conditions are indicated on the top. Antibodies used for immunoblot detection are depicted on the left

Renal cell carcinoma RCC FG1 cells [18] were used to search for nuclear interaction partners of galectin-3. In a first approach, NE from RCC FG1 cells were immunoprecipitated with anti-galectin-3 antibodies, and the co-precipitated proteins were separated by SDS-PAGE followed by colloidal coomassie staining of the gels and analyzed by mass spectrometry (data not shown). Here, the heterogeneous nuclear ribonucleoprotein A2B1 (hnRNPA2B1) was identified as a galectin-3 interacting protein (MASCOT score 106; sequence coverage 51.8 %). To confirm this result and to obtain a more comprehensive view of nuclear galectin-3 interaction partners, we employed a complementary approach. Recombinant human galectin-3 was coupled to a sepharose column. The column was loaded with NE from RCC FG1 cells, washed intensively and 150 mM lactose was added to release interaction partners that specifically bind to the lactose-free form of galectin-3 [26]. Eluted fractions were collected and analyzed by mass spectrometry. In addition to established interaction partners of galectin-3 both isoforms of hnRNPA2B1, isoform B1 and A2, were identified, (Fig. 1a and Additional file 1: Table S1). This observation confirmed the co-precipitation of hnRNPA2B1 with galectin-3. Moreover, additional hnRNP proteins and components of the splicing machinery including the splicing auxiliary factor U2AF65 were identified as galectin-3 interactors (Fig. 1a and Additional file 1: Table S1). We also verified the interaction between galectin-3 and hnRNPA2B1 or U2AF65 by co-immunoprecipitation (Fig. 1b, Additional file 2: Figure S1). Although we observed some unspecific binding of hnRNPA2B1 and U2AF65 to protein G beads, protein G beads loaded with galectin-3 antibodies precipitated significantly higher amounts of hnRNPA2B1 and U2AF65 (Fig. 1b, Additional file 2: Figure S1, lane 1).

hnRNPA2B1 is involved in pre-mRNA splicing and has been detected in the spliceosomal complex [27]. To test if the hnRNPA2B1/galectin-3 interaction is specific to RCC cells or if it can also be detected in other cell types we extended our analysis to HeLa cells. This cell line is an appropriate model system for our subsequent investigation of splicing effects, since the function of hnRNPA2B1 in pre-mRNA splicing in HeLa cells is well established [28, 29]. We thus performed blue native gel electrophoresis of HeLa NE to monitor the presence of hnRNPA2B1 and galectin-3 in nuclear protein complexes. At first, smaller polypeptides including galectin-3 monomers with a molecular weight of about 29 kDa were depleted from the NE using centricon filters with a cut-off molecular mass of 50 kDa. The remaining extracts were then separated sequentially on a native gradient gel (first dimension) followed by resolving the complexes according to their molecular weight by SDS-PAGE (second dimension). As assessed by immunoblot the distribution patterns of galectin-3 and hnRNPA2B1 along the native gradient are similar with one distinct maximum in the 10 % range of the first dimension, suggesting that both proteins co-reside in stable supramolecular assemblies or protein complexes (Fig. 1c).

Taken together, our biochemical characterization identifies hnRNPA2B1 as a novel interaction partner of galectin-3 and suggests that the two proteins form a complex in NE from RCC FG1 and Hela cells.

**Fig. 2:**
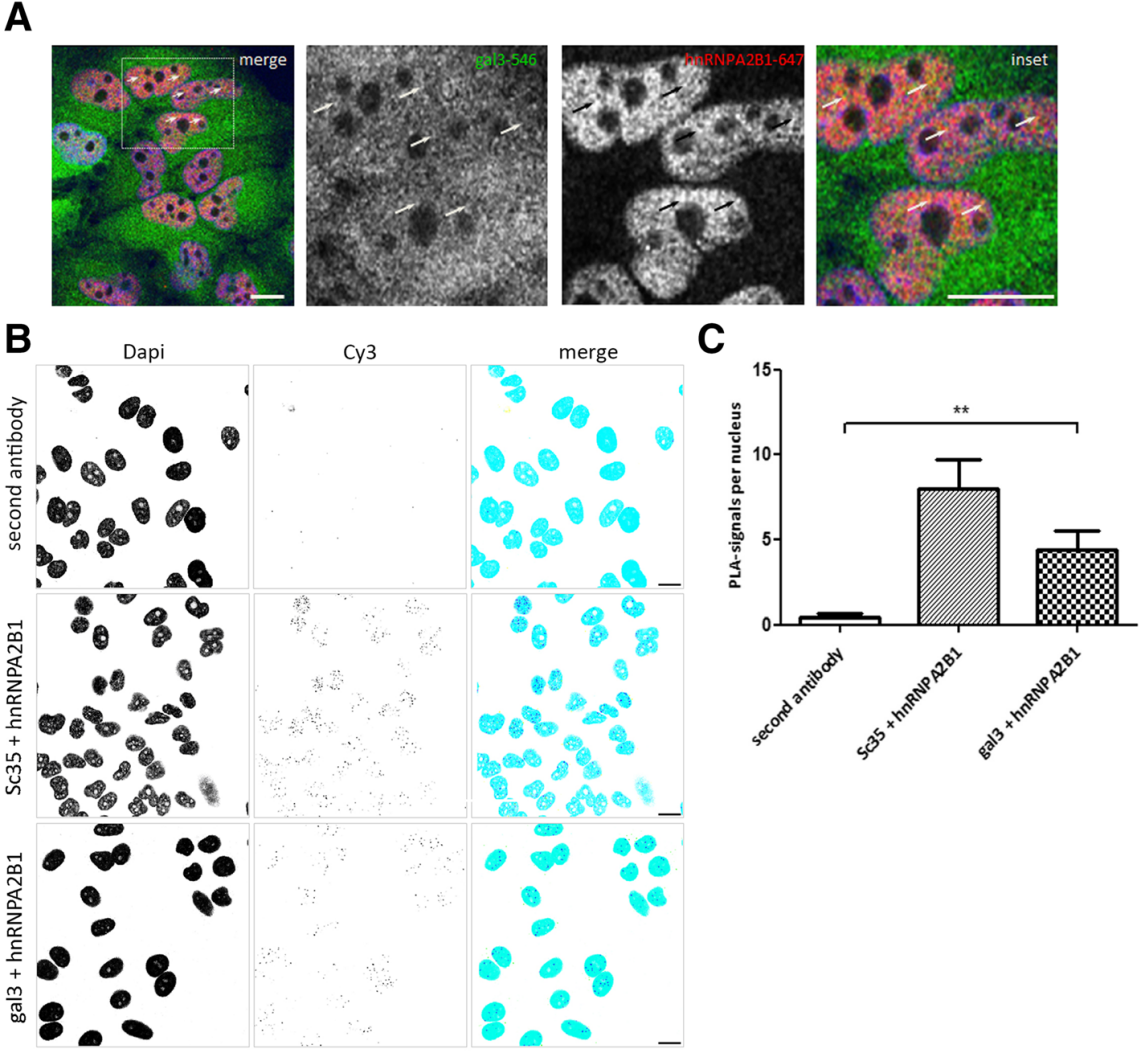
Localisation of galectin-3 and hnRNPA2B1 in cell nuclei. **a** HeLa cells were fixed and stained by immunofluorescence with anti-galectin-3/ Alexa Fluor 546 together with anti-hnRNPA2B1/ Alexa Fluor 647. Punctate structures positive for hnRNPA2B1 and endogenous galectin-3 are indicated by arrows. Nuclear staining (DAPI) is depicted in blue, scale bars: 10 μm. **b** Interaction between galectin-3 and hnRNPA2B1 was assessed by *in situ* PLA. HeLa cells were fixed and incubated with antibodies directed against galectin-3 and hnRNPA2B1. In the negative control HeLa cells were incubated in the absence of primary antibodies. HeLa cells were incubated with primary antibodies against hnRNPA2B1 and Sc35 as positive control. Interactions in a proximity up to 40 nm appear as fluorescent dots. Nuclei were stained with DAPI. For a better comparison, nuclei are depicted in blue, cytoplasmic PLA-signals in magenta and nuclear PLA-signals in dark blue in the merged images. Scale bars; 10 μm. **c** The amount of PLA-spots per nucleus was quantified. Bar graphs indicate the average relative number of PLA-signals per nucleus +/− SD, *n* = 3 (** *p* = 0.01)

We next addressed the question if hnRNPA2B1 and galectin-3 also colocalize in vivo by immunofluorescence microscopy. As depicted in Fig. 2a, hnRNPA2B1 showed strong nuclear staining, whereas galectin-3 was evenly distributed between cytoplasm and cell nuclei. In the merged images some of the brighter galectin-3-positive nuclear spots were also stained by antibodies directed against hnRNPA2B1, which indicates that both partner proteins accumulate within similar nuclear regions. Furthermore, spatial proximity of galectin-3 and hnRNPA2B1 was tested by employing the *in situ* PLA, which allows for improved detection of protein complexes [30]. In this assay close proximity between the epitopes of two distinct primary antibodies is required to allow the formation of amplifiable circularized ligation products. The amplicons were thereafter visualized with fluorescent detection probes as exemplified in the positive control of Fig. 2b. Here, primary antibodies against the two verified binding partners hnRNPA2B1 and SC35 were used [27]. PLA signals also appeared in the DAPI-stained nuclei with primary antibodies directed against galectin-3 and hnRNPA2B1. On the other hand, only a few faint spots were visible in the complete absence of primary antibodies as negative control. Quantification of PLA signals per cell nucleus revealed statistically significant numbers of these signals in the presence of primary antibodies directed against hnRNPA2B1 and galectin-3 (Fig. 2c). Thus indicating that hnRNPA2B1 and galectin-3 come into close proximity within Hela cell nuclei.

Altogether, our biochemical and fluorescence microscopy data suggest that hnRNPA2B1 interacts with galectin-3 in the cell nucleus. The idea that both partners are members of a spliceosomal complex is further corroborated by galectin-3-mediated pulldown of 13 hnRNP proteins and spliceosomal components as identified by mass spectrometry (Fig. 1a and Additional file 1: Table S1) or immunoblot/immunofluorescence analysis (Additional file 2: Figure S1).

### Galectin-3 in mRNA-spicing and -export

**Fig. 3:**
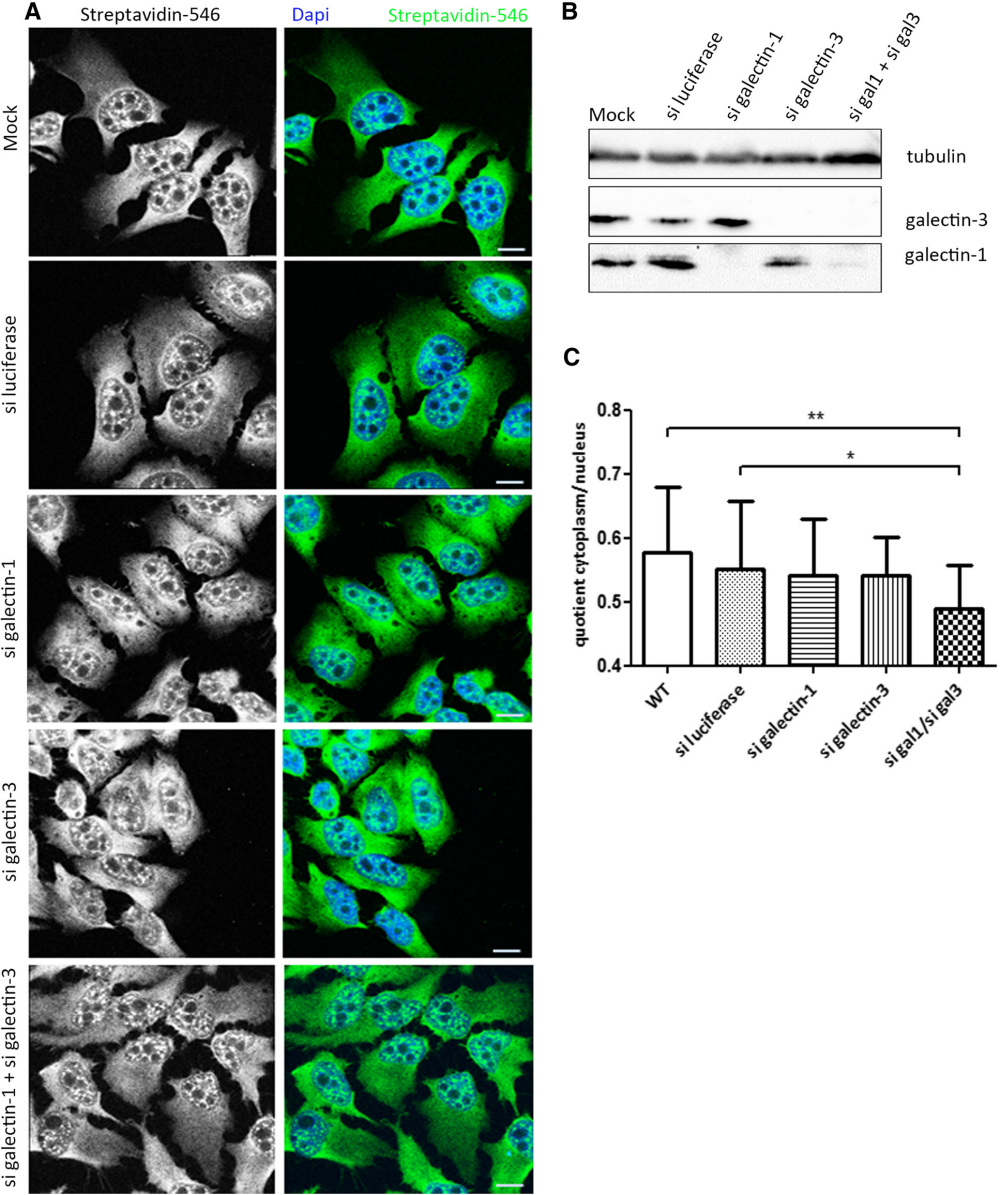
FISH analysis of Hela cells depleted of galectin-1 and/or galectin-3. **a** At first HeLa cells were transfected with siRNA to silence galectin-1, galectin-3 or both proteins. As a control for non-silencing siRNA, the cells were transfected with the firefly luciferase siRNA. Knockdown-efficiency of galectin-1 and/ or galectin-3 was assessed by immunoblot (**b**). The cells were fixed and the mRNA was stained with Biotin-oligo(dT)/ Streptavidin-Alexa Fluor 546. Nuclear staining (DAPI) is indicated in blue, scale bars: 10 μm. **c** Fluorescent mRNA-staining as depicted in (**a**) in the cytoplasm and the nucleus was quantified using the Leica LAS AF software package. Quotients of cytoplasmic divided by nuclear staining +/− SD are depicted. Statistical significant differences are indicated (*n* = 9, * *P* = 0.05 and ** *P* = 0.005)

It has previously been published that hnRNPA2B1 is involved in mRNA-splicing and -export (7) and we sought to assess the role of galectin-3 in these two processes by siRNA-mediated galectin-3 depletion. First, HeLa cells were transfected with galectin-3-specific siRNA and with luciferase-siRNA as a control (Fig. 3). Efficient depletion of galectin-3 was verified by immunoblot from cell lysates (Fig. 3b). To determine the mRNA distribution in the cell nuclei and in the cytoplasm, *in situ* hybridization (FISH) of mRNA with labelled oligo dT-probes was employed in galectin-3-depleted and control cells. Ratios of cytoplasmic and nuclear mRNA levels were quantified from confocal images (Fig. 3c). These data revealed no significant alterations in the subcellular mRNA distribution pattern following galectin-3 depletion. Since previous studies had shown that galectin-3 and galectin-1 exhibit functional redundancy in their splicing activity and nuclear localisation [31], we decided to knockdown galectin-1 and −3 simultaneously in Hela cells. This double knockdown displayed substantial nuclear mRNA-retention, while a single knockdown of galectin-1 had no significant implications on the mRNA distribution (Fig. 3c). Consequently, our data suggest that galectin-3 and galectin-1 contribute to efficient RNA processing and export in a redundant fashion.

**Fig. 4:**
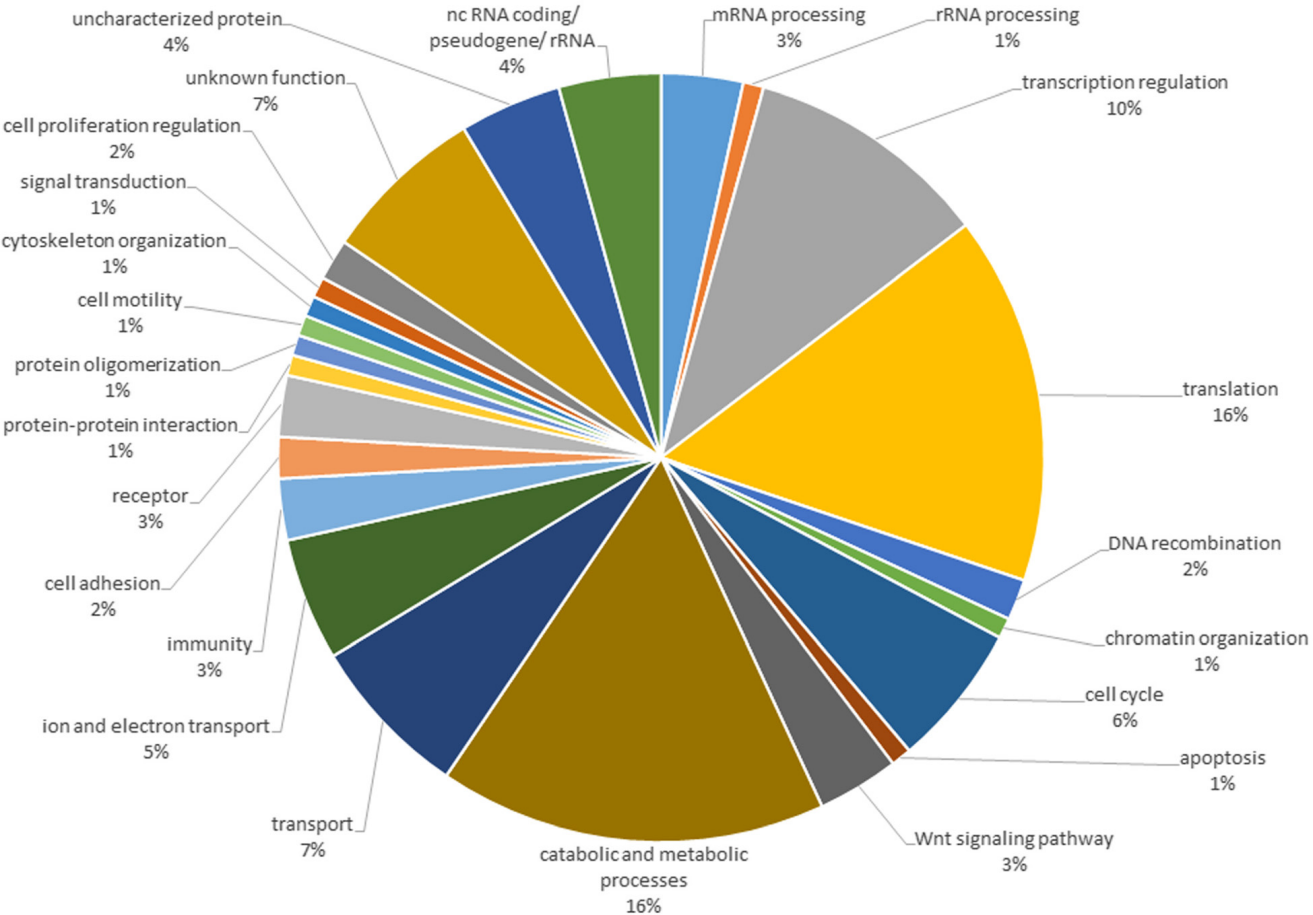
Influence of galectin-3 knockdown on pre-mRNA splicing of isoforms from specific transcripts. HeLa cells were transfected with siRNA to silence galectin-3 and with the non-silencing control luciferase. As assessed by RNA-sequencing transcripts alternatively spliced following galectin-3 depletion were sorted by function and shown in the diagram (*P* ≤ 0.05) (Additional file 4: Table S2)

Even if each of the two galectins can in general compensate the loss of one partner in RNA processing and export, we decided to search for more subtle galectin-3-specific effects. To receive a deeper insight into the specific involvement of galectin-3 in mRNA-splicing, we sequenced mRNAs from galectin-3 depleted and control HeLa-cells. Galectin-3-knockdown efficiency was verified by immunoblot (Additional file 3: Figure S2). RNA-seq libraries from these cells were sequenced via paired-end sequencing to obtain 2x50 bp paired reads. Gross changes in the mRNA expression pattern following galectin-3 depletion were not observed by RNA-seq data analysis, which is most likely due to a compensation by galectin-1 in galectin-3 depleted cells. Nevertheless, a detailed mRNA sequence analysis revealed statistically significant alterations in the splicing patterns of several genes. Especially, genes assigned to transcriptional and translational regulation, cell metabolism, intracellular transport or cell proliferation were affected (Fig. 4, Additional file 4: Table S2). These data are, at the level of mRNA-processing, in line with previous observations showing an involvement of galectin-3 in diverse cellular processes [2, 32]. To strengthen the functional link of galectin-3 and hnRNPA2B1 in a cancer context we searched for oncogenes that were similarly affected by galectin-3 and hnRNPA2B1 knockdown. This search identified the nuclear oncoprotein SET: As demonstrated by Goodarzi et al. specific knockdown of hnRNPA2B1 reduces the number of SET-transcripts [33]. Our RNA-seq data revealed that galectin-3 depletion significantly elevates a particularly processed form of SET-transcripts, which does not code for the protein (Additional file 4: Table S2). Thus, depletion of either of the two interaction partners reduces intracellular levels of SET.

## Discussion

Our data demonstrate that galectin-3 interacts with hnRNPA2B1 in the cell nucleus and that the presence of galectin-3 modulates mRNA-export and -splicing. Although we cannot formally rule out that the galectin-3 binding to hnRNPA2B1 is indirect our ability to detect galectin-3/hnRNPA2B1 complexes in different cell types using different biochemical approaches argues that this association is robust. It had already been demonstrated that hnRNPA2B1 is part of the splicing machinery and is involved in mRNA-processing [34, 35]. The hnRNP proteins A1, A2, B1 and B2, together with C1 and C2 assemble into particles to recruit the newly transcribed pre-mRNA into hnRNPs. They assemble into tetrameric (A2)_3_(B1)- or pentameric (A2)_3_(B1)(B2)-complexes in the particle-center with A1, C1 and C2 positioned peripherally [36]. This so-called H-complex of the splicing pathway has a regulatory function and influences the ability of particular RNAs to assemble productive splicing complexes. The H-complex is the first step before the initiation complex starts to recognize the initiation sites (E- and A-complex) on the pre-mRNA. Then, the B- and C-complex initiate the two transesterification reactions leading to exon-joining and intron-release (reviewed in [37]). Galectin-3 as interaction partner of hnRNPA2B1 in the H-complex would thus be involved in early steps of spliceosome assembly. This lectin had been already described as a factor that modulates the activity and formation of splicing complexes in HeLa cells [17]. It was also speculated that galectin-3 binds a common splicing partner through protein-protein interactions [38]. Furthermore, Wang et al. demonstrated that galectin-1 and galectin-3 are functionally redundant splicing factors as suggested earlier [31], and experiments with the C-terminal carbohydrate recognition domain of galectin-3 indicate that the amino-terminal domain of the lectin modulates splicing. Carbohydrates do not seem to be involved in the interaction of galectins with spliceosomal components as previously described for galectin-1 by Voss et al. [39]. Additional evidence for the presence of galectin-3 in spliceosomes comes from colocalisation experiments with the speckles-marker Sc35 [31] or the Sm epitopes of the small nuclear ribonucleoprotein complexes (snRNP) [40]. Moreover, galectin-3 sedimented in cesium sulfate gradients at densities consistent with the ones of hnRNPs and snRNPs [16]. These observations strongly suggest that galectin-3 is a member of the spliceosomal complex. However, the role and number of galectin-3-interaction-partners in this process is unclear. According to Wang et al. the pre-mRNA as binding partner could be excluded [38]. Haudek et al. described that about 70 % of nuclear galectin-3 is complexed in high molecular mass particles [41]. They identified the U1-specific protein, U1 70 K, of the E-complex as galectin-3 interaction partner. We have now found additional binding partners of galectin-3 by co-immunoprecipitation and mass spectrometry, which associate at an early stage of spliceosome assembly as members of the H-, E- and A-complex, most prominently hnRNPA2B1 from the H-complex. A putative role of galectin-3 in H-complex assembly comes from another study showing that addition of the N-terminal galectin-3-domain arrested pre-mRNA splicing at a position corresponding to the H-complex [42]. In that study, galectin-1 pulled down Gemin4. Fragments of Gemin4 also exhibited dominant negative effects when added to a cell-free splicing assay. In addition, galectin-1 co-immunoprecipitated galectin-3 from NE [42]. Our isolation of galectin-1 from galectin-3/ sepharose columns confirmed this observation. It thus seems likely that the two galectins are members of identical splicing complexes. Another hint for this idea comes from the functional redundancy of galectin-1 and −3 as described earlier for pre-mRNA splicing [31] and which is shown for mRNA-export by our study. RNA-splicing and nuclear export are directly linked to each other [43] so that a reduction in RNA-export efficiency may be due to alterations in the splicing machinery and/or to the assembly of nuclear RNA-binding factors in specifying the cytoplasmic fate of an RNA molecule.

The observation, that the number or processing of RNA-transcripts for the protein SET is affected by both galectin-3- as well as by hnRNPA2B1-depletion, points to specific functions of the nuclear galetin-3/hnRNPA2B1-complex in the regulation of SET expression. Interestingly, SET expression is deregulated in more than 10 % of kidney cancer samples [44], a cancer type where we have previously demonstrated increased nuclear translocation of galectin-3 [18]. SET is a key modulator in cell proliferation and interacts with hnRNPA2B1 [45]. Moreover, SET and hnRNPA2B1 are specific inhibitors of the tumor suppressor protein phosphatase 2A (PPA2), a major phosphatase that controls cell proliferation [45, 46]. Consequently, our results suggest that in addition to PPA2-inhibition, hnRNPA2B1 in complex with galectin-3 stimulates cell proliferation by increasing the number of protein coding SET-transcripts.

## Conclusions

As a conclusion, our data suggest that galectin-3 in association with hnRNPA2B1 is involved in the early assembly of the splicing machinery for mRNA-processing and nuclear export.

## Abbreviations

CRD, carbohydrate recognition domain; FISH, fluorescence *in situ* hybridization; HeLa, human cervix carcinoma cells; hnRNP, heterogenous nuclear ribonucleoprotein; mAb, monoclonal antibody; NE, nuclear extracts; PLA, proximity ligation assay; PPA2, protein phosphatase 2A; snRNP, small nuclear ribonucleoprotein complexes

## Additional files

Additional file 1: Table S1. Galectin‐3 interacting partners. (PDF 88 kb) Additional file 2: Figure S1. Localisation and interaction of galectin-3 and U2AF65. (A) Nuclear extracts from HeLa cells were immunoprecipitated with mAb anti-U2AF65. The precipitated proteins were analysed by immunoblot with antibodies directed against U2AF65 and galectin-3. Mock is the beads control without an antibody incubation. (B) HeLa cells were fixed and stained by immunoflourescence with anti-galectin-3/AlexaFlour 647 and anti-U2AF65/AlexaFlour 546. Structures positive for U2AF65 and endogenous galectin-3 are indicated by arrows. Nuclei (Hoechst 33342) are depicted in blue, scale bars: 10 μm. (JPG 1601 kb) Additional file 3: Figure S2. Galectin-3 knockdown verification for RNA-seq analysis. HeLa cells were transfected with siRNA to silence galectin-3 and luciferase as silencing control. The silencing effifiency was analysed by immunoblot with anti-galectin-3 and anti-tubulin antibodies as housekeeping control. (JPG 803 kb) Additional file 4: Table S2. Differentially spliced mRNAs following galectin‐3 depletion. (PDF 122 kb)
