# Supplementary material for: Galectin-3 interacts with components of the nuclear ribonucleoprotein complex
Source: BMC Cancer. 2016 Jul 19;16:502. doi: 10.1186/s12885-016-2546-0 (PMC4952364; doi:10.1186/s12885-016-2546-0)
Supplement: Additional file 1: Table S1. — Galectin‐3 interacting partners. (PDF 88 kb) [file 12885_2016_2546_MOESM1_ESM.pdf]

**Table S1**. **Galectin-3 interacting partners.**

| | Identifier | Mw KDa | Number of peptides | number of proteins | coverage (%) | Description |
|---|---|---|---|---|---|---|
| 1 | 13654237 | 468,8 | 71 | 15 | 25,68 | DNA-dependent protein kinase catalytic subunit isoform1 (DNPK1) |
| 2 | 119591368 | 58,5 | 17 | 15 | 40,49 | nucleolin, isoform CRA_C (Ncl) |
| 3 | 157412270 | 73,6 | 15 | 17 | 36,18 | hnRNPM isoform b |
| 4 | 14043072 | 37,4 | 13 | 9 | 44,19 | hnRNPA2B1 isoform B1 |
| 5 | 75517570 | 29,4 | 12 | 41 | 41,2 | hnRNPA1 |
| 6 | 154355000 | 73,1 | 11 | 15 | 23,07 | far upstream element-binding protein 2 |
| 7 | 24234750 | 95,3 | 11 | 48 | 20,36 | interleukin enhancer-binding factor 3 isoform a |
| 8 | 100913206 | 140,9 | 11 | 15 | 11,97 | ATP-dependent RNA helicase A |
| 9 | 157694492 | 148,8 | 10 | 13 | 12,58 | myb-binding protein 1A isoform 2 |
| 10 | 19913410 | 99,3 | 10 | 24 | 23,85 | major vault protein |
| 11 | 24234686 | 53,5 | 10 | 80 | 24,54 | heat shock cognate 71 kDa protein isoform 2 |
| 12 | 126032350 | 465,2 | 9 | 12 | 3,27 | DNA-dependent protein kinase catalytic subunit isoform2 |
| 13 | 14165466 | 57,2 | 9 | 15 | 34,65 | polypyrimidine tract-binding protein 1 isoform c (PTBP1) |
| 14 | 5031877 | 66,4 | 8 | 8 | 20,99 | lamin B1 |
| 15 | 40353734 | 29,4 | 8 | 34 | 46,04 | nucleophosmin isoform 2 |
| 16 | 4507555 | 75,4 | 8 | 8 | 18,3 | thymopoietin isoform alpha |
| 17 | 4507943 | 123,3 | 8 | 16 | 12,7 | exportin-1 |
| 18 | 5031753 | 49,2 | 8 | 29 | 27,84 | hnRNPH |
| 19 | 211828181 | 61,9 | 8 | 9 | 31,12 | hnRNPL |
| 20 | 116283697 | 50,6 | 8 | 20 | 26,49 | SYNCRIP (hnRNPQ) |
| 21 | 4505773 | 29,8 | 7 | 12 | 45,59 | prohibitin |
| 22 | 33875177 | 29,4 | 7 | 17 | 41,73 | YBX1 protein, partial |
| 23 | 5729877 | 70,9 | 7 | 60 | 15,33 | heat shock cognate 71 kDa protein isoform 1 |
| 24 | 4885381 | 22,6 | 6 | 2 | 22,57 | histone H1.5 |
| 25 | 4503101 | 20,9 | 6 | 3 | 45,6 | cysteine and glycine-rich protein 2 |
| 26 | 40254924 | 34,9 | 6 | 1 | 30,62 | leucin-rich repeat-containing protein 59 |
| 27 | 120587023 | 318,2 | 6 | 5 | 3,3 | small subunit processome component 20 homolog |
| 28 | 4826760 | 45,6 | 6 | 5 | 31,08 | hnRNPF |
| 29 | 4504447 | 36 | 6 | 8 | 24,63 | hnRNPA2B1 isoform A2 |
| 30 | 111494085 | 67,8 | 6 | 14 | 15,32 | hnRNPR |
| 31 | 30795231 | 22,7 | 5 | 4 | 52,42 | brain acid soluble protein 1 (BASP1) |
| 32 | 4885375 | 21,4 | 5 | 13 | 24,41 | histone H1.2 |
| 33 | 49257460 | 33,3 | 5 | 9 | 28 | PTRF protein |
| 34 | 224586882 | 40,1 | 5 | 16 | 20,7 | DNA-binding protein A isoform a (YBX3) |
| 35 | 4504517 | 22,8 | 5 | 10 | 40 | heat shock protein beta-1 |
| 36 | 9624998 | 49,2 | 5 | 6 | 18,26 | hnRNPH2 |
| 37 | 91208426 | 273,4 | 5 | 12 | 3 | pre-mRNA-splicing factor 8 (PRPF8) |
| 38 | 54112117 | 145,7 | 5 | 5 | 5,06 | SF3B1 isoform 1 |
| 39 | 55956921 | 30,6 | 5 | 23 | 15,79 | hnRNPAB isoform b |
| 40 | 5031875 | 65,1 | 4 | 25 | 12,06 | lamin isoform c |
| 41 | 433344 | 31,5 | 4 | 40 | 15,73 | p37 AUF 1 |
| 42 | 13128860 | 55,1 | 4 | 15 | 11,41 | histone deacetylase 1 (HDAC1) |
| 43 | 21626466 | 94,6 | 4 | 18 | 8,5 | matrin-3 isoform a |
| 44 | 300934782 | 97,3 | 4 | 12 | 6,17 | transportin-3 isoform 2 |
| 45 | 119573569 | 98,7 | 4 | 22 | 5,64 | adenosine deaminase, RNA-specific, isoform CRA_b (ADAR) |
| 46 | 62955803 | 195,9 | 4 | 11 | 3,26 | nucleoporin Nup 188 homolog |
| 47 | 295054210 | 271,5 | 4 | 10 | 2,78 | telomere-associated protein RIF1 isoform 2 (RIF1) |
| 48 | 301171475 | 71,3 | 4 | 14 | 8,36 | DDX3X isoform 3 |
| 49 | 4502491 | 31,3 | 4 | 10 | 29,08 | SF2P32 |
| 50 | 21536301 | 83 | 3 | 13 | 6,18 | signal transducer & activator of transcription 1-isoform beta (STAT1) |
| 51 | 338753429 | 79,8 | 3 | 9 | 5,6 | nuclear pore complex protein Nup93 isoform 2 |
| 52 | 148596949 | 73,6 | 3 | 6 | 5,15 | nucleolar and coiled-body phosphoprotein 1 (NOLC1) |
| 53 | 11225260 | 90,7 | 3 | 19 | 4,84 | DNA-topoisomerase 1 |
| 54 | 4504981 | 14,7 | 3 | 11 | 42,22 | galectin-1 |
| 55 | 48429165 | 26,9 | 3 | 5 | 26,85 | Tho4 |
| 56 | 118582269 | 22,4 | 3 | 11 | 26,37 | serine/arginine-rich splicing factor 1 isoform 2 (SRSF1) |
| 57 | 52632385 | 50,5 | 3 | 9 | 14,47 | hnRNPL isoform b |
| 58 | 197692223 | 53,1 | 3 | 12 | 11,68 | U2 auxiliary factor 2 isoform b (U2AF65) |
| 59 | 119620769 | 15,2 | 3 | 10 | 30,3 | splicing factor, arginine/serine-rich 7, 35 kDa, isoform CRA_b (SRSF7) |
| 60 | 10800138 | 13,9 | 2 | 31 | 19,05 | histone H2B type 1-D |
| 61 | 4758302 | 12,3 | 2 | 2 | 30,77 | enhancer of rudimentary homolog (ERH) |

| | | | | | | |
|---|---|---|---|---|---|---|
| 62 | 83641870 | 28,4 | 2 | 27 | 16,22 | nucleophosmin isoform 3 |
| 63 | 4506007 | 37 | 2 | 33 | 9,29 | ser/thr-phosphatase PP1-gamma catalytic subunit isoform 1 (PPP1CC) |
| 64 | 5453555 | 24,4 | 2 | 14 | 8,8 | GTP-binding nuclear protein Ran |
| 65 | 4507353 | 61,5 | 2 | 20 | 6,96 | TATA-binding protein-associated factor 2N isoform 2 (TAF15) |
| 66 | 4758844 | 148,9 | 2 | 7 | 1,58 | nuclear pore complex protein Nup155 isoform 2 |
| 67 | 5174449 | 22,5 | 2 | 2 | 10,33 | histone H1x |
| 68 | 4504433 | 10,7 | 2 | 4 | 19,79 | high mobility group protein HMG-1/HMG-Y isoform b (HMGA1) |
| 69 | 4504183 | 23,3 | 2 | 27 | 18,57 | glutathione S-transferase P |
| 70 | 13376840 | 33,6 | 2 | 7 | 12,13 | WD repeat-containing protein 61 (WDR61) |
| 71 | 296439283 | 227,8 | 2 | 9 | 1,29 | nuclear pore complex protein Nup205 |
| 72 | 40217847 | 244,4 | 2 | 13 | 1,59 | U5 200 kDa (snRNP200) |
| 73 | 7657381 | 55,1 | 2 | 2 | 7,54 | PRP 19 |
| 74 | 402794208 | 54 | 2 | 17 | 7,62 | PUF60 isoform h |
| 75 | 7661920 | 46,8 | 2 | 26 | 9,98 | eIF4A3 |
| 76 | 197692189 | 27,9 | 2 | 5 | 12,92 | U2 auxiliary factor 1 isoform a (U2AF35) |
| 77 | 4504517 | 22,8 | 2 | 9 | 13,17 | HspB1 |
| 78 | 345197228 | 21,9 | 2 | 11 | 19,15 | Tra2B isoform 2 |
| 79 | 119577432 | 84,4 | 2 | 12 | 4,65 | hnRNP U-like 1, isoform CRA_c |
| 80 | 39753957 | 66,2 | 1 | 7 | 2,06 | torsin-1A-interacting protein 1 isoform 2 |
| 81 | 13775200 | 10,1 | 1 | 1 | 17,44 | SF3B5 |
| 82 | 31455228 | 19,8 | 1 | 5 | 10,78 | U2 auxiliary factor 1 (U2AF35) |
| 83 | 4507129 | 10,8 | 1 | 4 | 11,96 | small nuclear ribonucleoprotein E (SmE) |
| 84 | 5803036 | 30,8 | 1 | 2 | 6,56 | hnRNPA0 |

Binding partners of galectin-3 were eluted with lactose from a galectin-3/sepharose column and analyzed by mass spectrometry. Galectin-3 interacting proteins verified by co-immunoprecipitation and immunofluorescence analysis are indicated in red. Other hnRNPs are depicted in blue and components of the spliceosome in green.
