# Supplementary figures and images for: Galectin-3 interacts with components of the nuclear ribonucleoprotein complex
Source: BMC Cancer. 2016 Jul 19;16:502. doi: 10.1186/s12885-016-2546-0 (PMC4952364; doi:10.1186/s12885-016-2546-0)

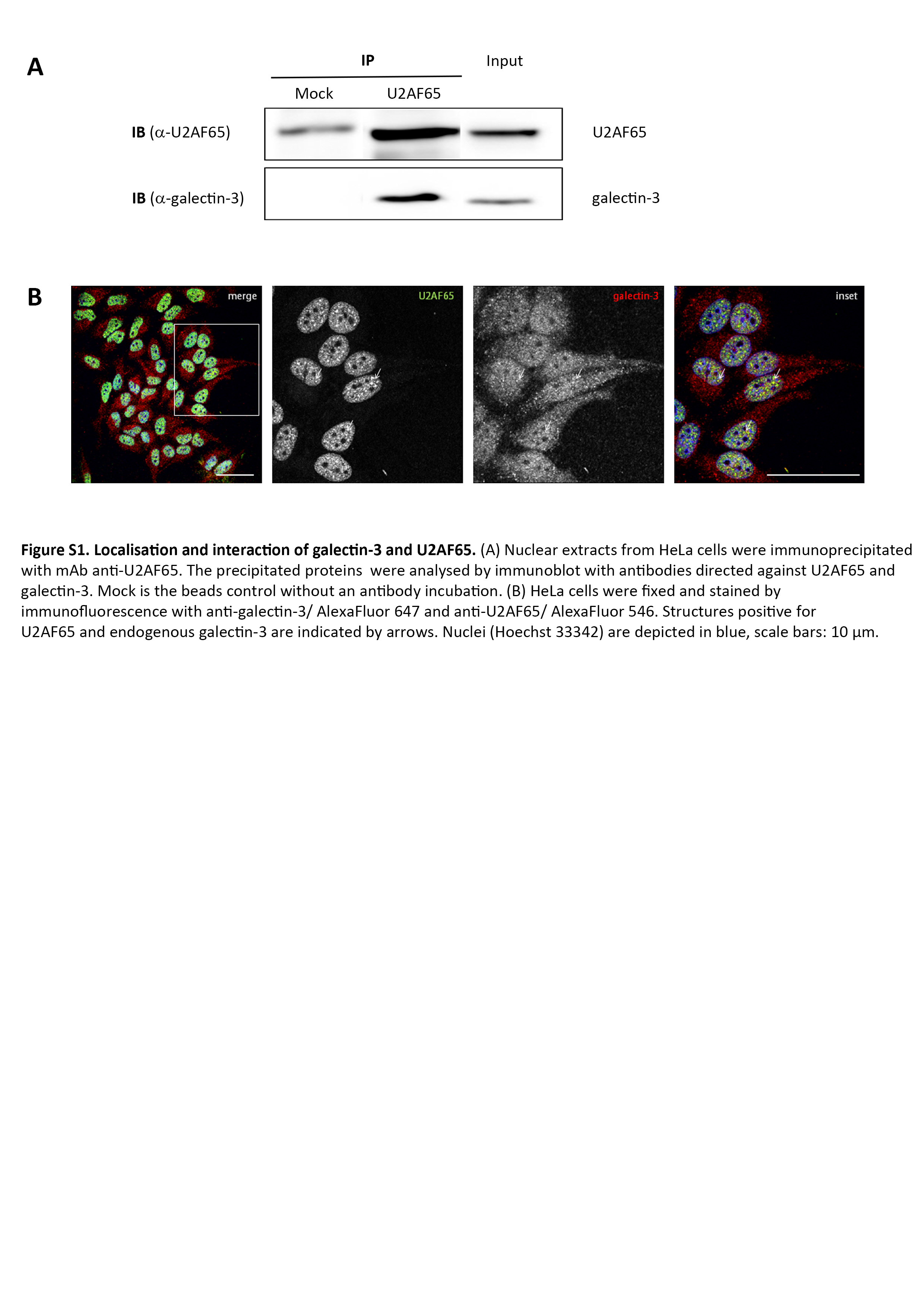

Supplement: Additional file 2: Figure S1. — Localisation and interaction of galectin-3 and U2AF65. (A) Nuclear extracts from HeLa cells were immunoprecipitated with mAb anti-U2AF65. The precipitated proteins were analysed by immunoblot with antibodies directed against U2AF65 and galectin-3. Mock is the beads control without an antibody incubation. (B) HeLa cells were fixed and stained by immunoflourescence with anti-galectin-3/AlexaFlour 647 and anti-U2AF65/AlexaFlour 546. Structures positive for U2AF65 and endogenous galectin-3 are indicated by arrows. Nuclei (Hoechst 33342) are depicted in blue, scale bars: 10 μm. (JPG 1601 kb) [file 12885_2016_2546_MOESM2_ESM.jpg]

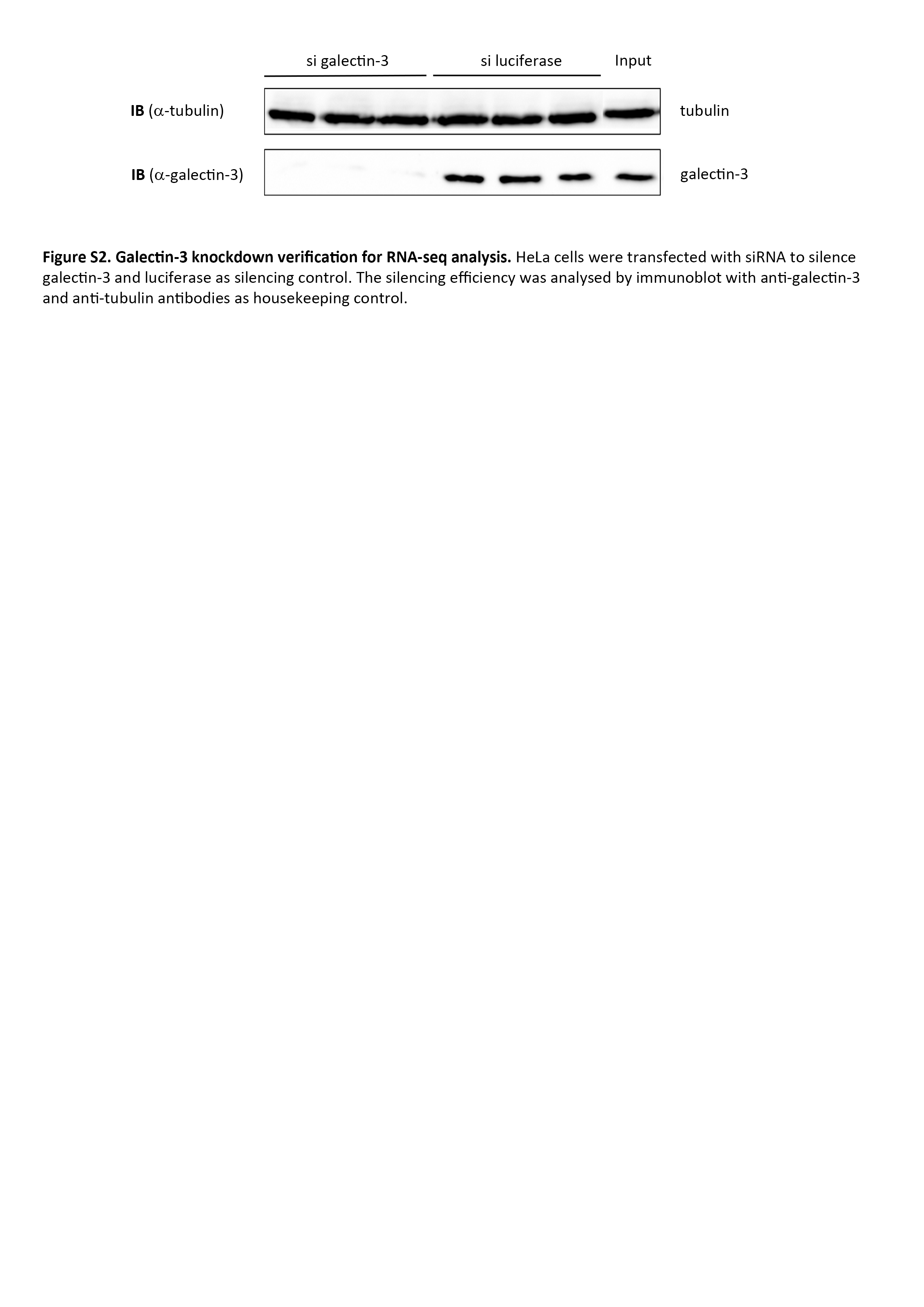

Supplement: Additional file 3: Figure S2. — Galectin-3 knockdown verification for RNA-seq analysis. HeLa cells were transfected with siRNA to silence galectin-3 and luciferase as silencing control. The silencing effifiency was analysed by immunoblot with anti-galectin-3 and anti-tubulin antibodies as housekeeping control. (JPG 803 kb) [file 12885_2016_2546_MOESM3_ESM.jpg]
