# Supplementary material for: Galectin-3 interacts with components of the nuclear ribonucleoprotein complex
Source: BMC Cancer. 2016 Jul 19;16:502. doi: 10.1186/s12885-016-2546-0 (PMC4952364; doi:10.1186/s12885-016-2546-0)
Supplement: Additional file 4: Table S2. — Differentially spliced mRNAs following galectin‐3 depletion. (PDF 122 kb) [file 12885_2016_2546_MOESM4_ESM.pdf]

Table S2. Differentially spliced mRNAs following galectin-3 depletion.

stable_id	padj	transcripts	gene name
ENSG00000005893	0,02173585	ENST00000540603	LAMP2
ENSG00000022840	0,0498587	ENST00000537376	RNF10
ENSG00000047315	0,03048084	ENST00000381227	POLR2B
ENSG00000047315	0,00960713	ENST00000381227	POLR2B
ENSG00000054523	0,0064032	ENST00000497835	KIF1B
ENSG00000063046	0,01240177	ENST00000550390	EIF4B
ENSG00000063177	0,00271401	ENST00000550671	RPL18
ENSG00000063177	0,00606828	ENST00000550671	RPL18
ENSG00000070756	0,02128759	ENST00000517990	PABPC1
ENSG00000071564	0,00447609	ENST00000586164	TCF3
ENSG00000072042	0,01322597	ENST00000557331	RDH11
ENSG00000075624	0,0163336	ENST00000425660	ACTB
ENSG00000078304	0,03281571	ENST00000556493	PPP2R5C
ENSG00000079841	0,01117241	ENST00000517433	RIMS1
ENSG00000079841	0,00830955	ENST00000517433	RIMS1
ENSG00000079841	0,01780655	ENST00000517433	RIMS1
ENSG00000079841	0,0069257	ENST00000517433	RIMS1
ENSG00000081181	0,00060702	ENST00000557120	ARG2
ENSG00000087077	0,00189214	ENST00000437505	TRIP6
ENSG00000089157	0,00387036	ENST00000546989	RPLP0
ENSG00000089157	0,03040067	ENST00000546989	RPLP0
ENSG00000089157	0,01981512	ENST00000546989	RPLP0
ENSG00000089157	0,02445019	ENST00000546989	RPLP0
ENSG00000092853	0,03348098	ENST00000520551	CLSPN
ENSG00000096746	4,95E-08	ENST00000354695	HNRNPH3
ENSG00000089157	1,48E-05	ENST00000546989	RPLP0
ENSG00000089157	2,08E-06	ENST00000546989	RPLP0
ENSG00000099622	0,01989357	ENST00000590171	CIRBP
ENSG00000103018	0,0017749	ENST00000512062	CYB5B
ENSG00000105968	0,00243361	ENST00000381124	H2AFV
ENSG00000108788	5,56E-07	ENST00000590050	MLX
ENSG00000109475	0,01894095	ENST00000394668	RPL34
ENSG00000109475	0,0163336	ENST00000394668	RPL34
ENSG00000109475	2,37E-12	ENST00000394668	RPL34
ENSG00000109685	1,66E-06	ENST00000382895	WHSC1
ENSG00000109861	0,04115452	ENST00000529974	CTSC
ENSG00000111640	0,0163336	ENST00000229239	GAPDH
ENSG00000112245	9,37E-05	ENST00000578299	PTP4A1
ENSG00000113552	0,04627731	ENST00000504139	GNPDA1
ENSG00000115252	0,03846657	ENST00000358139	PDE1A
ENSG00000117868	0,02291362	ENST00000435514	ESYT2
ENSG00000113719	0,03999813	ENST00000519796	ERGIC1
ENSG00000119048	0,02173585	ENST00000511807	UBE2B
ENSG00000119335	9,64E-05	ENST00000480217	SET
ENSG00000120256	0,04005437	ENST00000463728	LRP11
ENSG00000120685	0,01117241	ENST00000602899	PROSER1
ENSG00000120685	0,00274453	ENST00000602899	PROSER1

ENSG00000123080	0,00030463	ENST00000371761	CDKN2C
ENSG00000124243	0,00089011	ENST00000485049	BCAS4
ENSG00000127540	0,00541498	ENST00000589880	UQCR11
ENSG00000129625	0,00319942	ENST00000504247	REEP5
ENSG00000130558	0,00023207	ENST00000539529	OLFM1
ENSG00000130558	0,01884108	ENST00000539529	OLFM1
ENSG00000130826	0,01622641	ENST00000475966	DKC1
ENSG00000131069	0,04285654	ENST00000480978	ACSS2
ENSG00000131470	0,00153715	ENST00000589505	PSMC3IP
ENSG00000131473	0,04537347	ENST00000590770	ACLY
ENSG00000132199	4,82E-05	ENST00000578647	ENOSF1
ENSG00000132341	0,00014559	ENST00000464211	RAN
ENSG00000132846	0,00830955	ENST00000505685	ZBED3
ENSG00000133112	0,01045759	ENST00000533567	TPT1
ENSG00000134748	0,02072701	ENST00000487160	PRPF38A
ENSG00000136938	0,00086202	ENST00000486769	ANP32B
ENSG00000138035	0,03230769	ENST00000260604	PNPT1
ENSG00000138326	0,0163336	ENST00000465692	RPS24
ENSG00000138326	0,02699893	ENST00000465692	RPS24
ENSG00000142089	0,03407771	ENST00000526811	IFITM3
ENSG00000142541	0,00197168	ENST00000472481	RPL13A
ENSG00000142541	0,04568049	ENST00000472481	RPL13A
ENSG00000142676	0,04537347	ENST00000482370	RPL11
ENSG00000143641	0,00711084	ENST00000366672	GALNT2
ENSG00000145592	0,04797616	ENST00000504562	RPL37
ENSG00000145592	0,03281571	ENST00000504562	RPL37
ENSG00000145730	0,00098568	ENST00000506260	PAM
ENSG00000147687	0,00192782	ENST00000522810	TATDN1
ENSG00000149136	0,02406436	ENST00000526696	SSRP1
ENSG00000149925	0,00192782	ENST00000562679	ALDOA
ENSG00000149925	0,04446963	ENST00000562679	ALDOA
ENSG00000155755	0,00169529	ENST00000286196	TMEM237
ENSG00000159131	0,04209808	ENST00000441403	GART
ENSG00000160991	0,024743	ENST00000488996	ORAI2
ENSG00000161010	0,01240177	ENST00000520698	C5orf45
ENSG00000161960	0,03407771	ENST00000581384	EIF4A1
ENSG00000161970	0,04603954	ENST00000584164	RPL26
ENSG00000161970	0,03021406	ENST00000584164	RPL26
ENSG00000161970	0,03368222	ENST00000584164	RPL26
ENSG00000161970	0,02927271	ENST00000584164	RPL26
ENSG00000163291	0,03394755	ENST00000512299	PAQR3
ENSG00000163597	2,65E-07	ENST00000493536	SNHG16
ENSG00000163946	0,03394755	ENST00000478052	FAM208A
ENSG00000164032	0,0230666	ENST00000529158	H2AFZ
ENSG00000164855	0,00456324	ENST00000449955	TMEM184A
ENSG00000165609	0,03342627	ENST00000378927	NUDT5
ENSG00000166130	0,01488349	ENST00000420861	IKBIP
ENSG00000166164	0,0019218	ENST00000475877	BRD7
ENSG00000166441	0,00086202	ENST00000314138	RPL27A
ENSG00000166441	0,03092736	ENST00000314138	RPL27A

ENSG00000166741	0,00023309	ENST00000542647	NNMT
ENSG00000167526	0,02173585	ENST00000472354	RPL13
ENSG00000167526	0,00189436	ENST00000472354	RPL13
ENSG00000167526	0,00024114	ENST00000472354	RPL13
ENSG00000167552	0,01780655	ENST00000295766	TUBA1A
ENSG00000167552	0,00619385	ENST00000295766	TUBA1A
ENSG00000168542	0,01462764	ENST00000467886	COL3A1
ENSG00000168610	0,04561908	ENST00000588065	STAT3
ENSG00000169851	0,03758912	ENST00000361762	PCDH7
ENSG00000169955	1,48E-05	ENST00000252799	ZNF747
ENSG00000173442	0,01061915	ENST00000533237	EHBP1L1
ENSG00000173786	0,00086202	ENST00000393888	CNP
ENSG00000175061	0,03048084	ENST00000580180	C17orf76-AS1
ENSG00000177954	0,00016062	ENST00000392558	RPS27
ENSG00000182054	0,03348098	ENST00000559482	IDH2
ENSG00000182372	0,00197168	ENST00000524258	CLN8
ENSG00000184014	0,01240177	ENST00000529977	DENND5A
ENSG00000187109	0,00185846	ENST00000552147	NAP1L1
ENSG00000187109	0,01117241	ENST00000552147	NAP1L1
ENSG00000187260	0,03394755	ENST00000334493	WDR86
ENSG00000187514	1,48E-05	ENST00000341369	PTMA
ENSG00000188343	0,00084753	ENST00000522803	FAM92A1
ENSG00000188846	0,03348098	ENST00000416518	RPL14
ENSG00000188846	0,04754824	ENST00000416518	RPL14
ENSG00000188895	0,04115452	ENST00000581246	MSL1
ENSG00000189306	0,00711084	ENST00000483303	RRP7A
ENSG00000196428	0,03696615	ENST00000480589	TSC22D2
ENSG00000197111	0,04710537	ENST00000548933	PCBP2
ENSG00000197111	0,00959888	ENST00000548933	PCBP2
ENSG00000198755	0,01117241	ENST00000464112	RPL10A
ENSG00000205542	0,00218405	ENST00000380636	TMSB4X
ENSG00000205542	0,04539845	ENST00000380636	TMSB4X
ENSG00000211448	0,00551967	ENST00000553594	DIO2
ENSG00000211460	0,04115452	ENST00000536142	TSN
ENSG00000213923	0,00020474	ENST00000451964	CSNK1E
ENSG00000213923	0,0230666	ENST00000451964	CSNK1E
ENSG00000213923	0,00456324	ENST00000451964	CSNK1E
ENSG00000214182	0,04115452	ENST00000607242	PTMAP5
ENSG00000221983	0,00052147	ENST00000595683	UBA52
ENSG00000242259	0,03048084	ENST00000509549	C22orf39
ENSG00000245532	0,00122219	ENST00000601801	NEAT1
ENSG00000250232	0,01989357	ENST00000376358	AF196779.12
ENSG00000258891	0,04568049	ENST00000555916	RP5-1021I20.5
ENSG00000258964	0,04603954	ENST00000555937	RP11-618G20.1
ENSG00000260916	0,01583578	ENST00000569205	CCPG1
ENSG00000261459	0,03345417	ENST00000569360	ZNF747
ENSG00000263809	0,03281571	ENST00000582471	RP11-849F2.7
ENSG00000265500	0,0230666	ENST00000579777	SENP3-EIF4A1
ENSG00000265500	0,04590137	ENST00000579777	SENP3-EIF4A1
ENSG00000265500	0,01117241	ENST00000579777	SENP3-EIF4A1

ENSG00000265500	0,0185882	ENST00000579777	SENP3-EIF4A1
ENSG00000266658	0,00029035	ENST00000579027	RNA28S5
ENSG00000268083	0,03646614	ENST00000594769	AC104534.3
ENSG00000268083	0,02817458	ENST00000594769	AC104534.3
ENSG00000268173	0,00154597	ENST00000593731	PIK3R2
ENSG00000268173	0,00897405	ENST00000593731	PIK3R2
ENSG00000268173	0,03457181	ENST00000593731	PIK3R2
ENSG00000268173	0,00154597	ENST00000593731	PIK3R2
ENSG00000268173	5,91E-06	ENST00000593731	PIK3R2
ENSG00000268173	1,48E-05	ENST00000593731	PIK3R2
ENSG00000268173	0,00041371	ENST00000593731	PIK3R2
ENSG00000268173	2,45E-05	ENST00000593731	PIK3R2
ENSG00000268173	0,01932015	ENST00000593731	PIK3R2
ENSG00000270800	0,04627731	ENST00000605528	RPS10-NUDT3

Transcripts showing differently spliced mRNAs in galectin-3-silenced HeLa cells in comparison to the non-silenced luciferase control using DEXseq (padj $\leq$ 0.05).
